# Association Between Dietary Soy Isoflavones Intake and the Risk of Hyperemesis Gravidarum: A Cross-Sectional Study in Chinese Pregnant Women

**DOI:** 10.3390/nu17071282

**Published:** 2025-04-07

**Authors:** Siyang Chen, Xinyu Zhang, Lan Zhang, Wenjie Cheng, Yuan Jin, Qian Ma, Le Ma, Shunming Zhang, Jing Lin

**Affiliations:** 1 School of Public Health, Xi’an Jiaotong University Health Science Center, Xi’an 710061, China; chensiy@stu.xjtu.edu.cn (S.C.); zhangxinyuzc@163.com (X.Z.); zhanglan@stu.xjtu.edu.cn (L.Z.); chengwj20@163.com (W.C.); jinyuan0828@stu.xjtu.edu.cn (Y.J.); maqian9871@163.com (Q.M.); 2 Key Laboratory for Disease Prevention and Control and Health Promotion of Shaanxi Province, Xi’an Jiaotong University, Xi’an 710061, China

**Keywords:** dietary soy isoflavones, soy isoflavones, legumes, soybeans, hyperemesis gravidarum

## Abstract

(1) Background: Diet plays a crucial role in the intake of phytoestrogens, which are closely related to the pathogenesis of some pregnancy complications. However, no studies have explored the potential association between soy isoflavones, a type of phytoestrogen, and the risk of hyperemesis gravidarum (HG). This study aims to investigate the correlation between dietary intake of soy isoflavones and the risk of HG. (2) Methods: As part of the China Birth Cohort Study (CBCS), 2418 pregnant Chinese women (mean age: 31.2 ± 3.4 years) were enrolled between April 2021 and September 2022. Dietary intake was evaluated using a validated 108-item semi-quantitative food frequency questionnaire, with soy isoflavones intake estimated based on five food groups. HG was defined as a condition characterized by a pregnancy-specific vomiting score (PUQE) ≥ 13, weight loss of ≥5% due to severe nausea and vomiting before 16 weeks of gestation, inability to eat or drink normally, significant limitations in daily activities due to severe nausea or vomiting, or the need for hospitalization caused by the condition. The association between soy isoflavones intake and HG was analyzed using binary logistic regression and restricted cubic spline regression. (3) Results: Among all participants, 212 women (8.8%) were diagnosed with HG. The dietary intake of soy isoflavones was 14.56 (IQR: 9.89, 25.36) mg/d. After full adjustment for confounding factors, the results indicated that individuals with the highest dietary intake of soy isoflavones had a lower risk of developing HG (OR: 0.56, 95% CI: 0.36, 0.88. *P*_trend_ = 0.012). (4) Conclusions: Higher dietary intake of soy isoflavones is associated with a reduced risk of HG. We advocate for a dietary approach to the management of HG that prioritizes the intake of legume-rich foods, particularly those abundant in soy isoflavones.

## 1. Introduction

Hyperemesis gravidarum (HG) refers to severe nausea and vomiting of pregnancy (NVP) that occurs during early pregnancy, symptoms are severe enough to prevent normal eating and drinking, significantly restrict daily activities, and may require hospitalization for treatment [1]. The global incidence of HG ranges from 0.3% to 10.8% [2]. Increasing evidence suggests that HG is associated with an elevated risk of maternal health complications, such as gestational hypertension, anemia, and eclampsia [3], as well as adverse birth outcomes, including preterm birth, low birth weight, and fetal growth restriction [4,5]. In extreme instances, the condition may lead to Wernicke’s encephalopathy, posing a direct threat to maternal health and life [6]. Therefore, the identification of potential risk factors that contribute to the onset and progression of HG is imperative for effective prevention strategies.

Estrogen, produced by the corpus luteum, has been associated with an increased risk of NVP [7]. While the production of estrogen by the corpus luteum is a normal physiological process, the variability in individual responses raises the question of why HG manifests in some individuals and not in others. Studies suggest that excessively high estrogen levels or heightened sensitivity to estrogen may be significant contributors to the severe vomiting observed in HG patients [8]. During pregnancy, maternal estradiol levels increase significantly, especially during the early to mid-pregnancy phases [9]. This hormone surge levels may exacerbate the clinical manifestations of HG via several mechanisms: firstly, estrogen may act on gastrointestinal smooth muscle, thereby reducing gastric emptying rate, which could exacerbate nausea and vomiting. Secondly, estrogen may influence dopamine and serotonin pathways, both of which are integral to the regulation of nausea and vomiting [10]. A randomized controlled trial (RCT) conducted in the United States involving 436 participants revealed that varying doses of estrogen preparations led to a persistent and significant increase of approximately 50% in the incidence of nausea and vomiting among those receiving higher doses of estrogen [11]. Therefore, it is theoretically plausible that a pharmacological agent capable of antagonizing or partially mitigating the effects of estrogen could alleviate clinical symptoms of HG. Natural estrogen antagonists found in soy-based foods have demonstrated the ability to counteract estrogen activity, potentially reducing the risk of estrogen-related conditions such as breast cancer, uterine fibroids, and menopausal symptoms [12,13,14]. However, the current literature lacks conclusive evidence regarding the efficacy of dietary soy isoflavones in ameliorating HG symptoms through antagonism, leaving the relationship between soy isoflavones intake and HG inadequately explored.

Therefore, we conducted a study to examine the association between dietary intake of soy isoflavones and the risk of HG, aiming to explore whether soy isoflavones consumption is associated with the risk of HG. This study may provide epidemiological evidence for future research on HG and the formulation of relevant health recommendations regarding HG.

## 2. Materials and Methods

### 2.1. Study Participants

This is a cross-sectional study utilizing data from the China Birth Cohort Study (CBCS), with data collected from pregnant women during their first prenatal visit between April 2021 and September 2022. The study explores the potential links between maternal nutrition, lifestyle factors, medication use, and health outcomes in both mothers and their children. The study design and detailed information have been previously reported [15,16]. Pregnant women aged 18–49 years at the time of their first prenatal care visit were enrolled in the cohort. Upon enrollment, a structured electronic questionnaire was used to collect data on sociodemographic variables, lifestyle factors, and anthropometric measurements, and obstetric-related information was obtained through the hospital’s Hospital Information System (HIS). The study participants were recruited from the Northwest Women and Children’s Hospital from April 2021 to September 2022. The inclusion criteria were: (1) age ≥ 18 years, gestational age between 6 and 16 weeks, and voluntary consent to participate in the study; (2) participants needed to be able to complete the questionnaire in Chinese. Women with cognitive or psychiatric disorders or those experiencing nausea and vomiting due to other medical conditions were excluded.

The sample size calculation was performed using the formula proposed by Cross et al., $n=\frac{Z^{2}\cdot p\cdot (1-p)}{E^{2}}$ [17]. Assuming the prevalence of HG (p) is 10.8%, the allowable error (E) is 2%, and the Type I error rate (α) is 0.01, the corresponding Z value is 2.576. The required sample size for this analysis was approximately 1597. To minimize potential bias from missing data and non-response, a total of 3122 women were recruited for the study. Among them, 607 participants did not complete all survey questionnaires and were excluded from the analysis. Additionally, 97 participants had partially missing data. In total, 704 (22.6%) participants were excluded due to incomplete information, leaving a final analytic sample of 2418 women. All participants gave written informed consent, and the study received approval from the Ethics Committee of the School of Medicine at Xi’an Jiaotong University (Approval No. 2020-1263).

### 2.2. Dietary Assessment

The dietary intake of the pregnant participants was assessed using an enhanced semi-quantitative Food Frequency Questionnaire (FFQ) comprising 10 categories and 108 items [18,19]. To capture long-term dietary habits beyond the pregnancy period, participants were instructed to report their typical consumption patterns over the preceding year, specifying the frequency of food servings on a monthly, weekly, or daily basis. This recall period spanned from the time of the survey, when participants were approximately 6 to 16 weeks pregnant back to the previous 12 months. The FFQ provided a food portion size guide, detailing the weight of each serving to help assess individual food intake ranges. Daily food intake was calculated based on the reported frequency of servings per day and the weight of each serving. Total daily energy intake was derived from the FFQ data in combination with the 2018 China Food Composition Table (Book 1, 2nd Edition).

### 2.3. Estimation of Dietary Soy Isoflavones

The calculation of dietary soy isoflavones intake included five food groups: legumes, tofu and its products, bean sprouts, soy milk, and certain vegetables and fruits containing minimal amounts of soy isoflavones. The legume group included seven varieties (soybeans, yellow soybeans, mung beans, black beans, green beans, red beans, and broad beans), while the tofu and its products group included five forms (tofu, rice tofu, dried tofu, tofu skin, and vegetarian chicken), the bean sprouts group included two types (mung bean sprouts and soybean sprouts), the soy milk group included three types (soybean milk, soy milk, and soy protein beverages), alongside 17 vegetables and fruits with relatively low levels of soy isoflavones. By using the daily intake reported in the FFQ for each food and the weight per serving, we calculated the concentrations of daidzein, genistein, and glycitein per 100 g of each food. The total intake of these three isoflavones was then aggregated to estimate the overall dietary soy isoflavones intake among pregnant women. It is noteworthy that the intake of any additional isoflavones supplements at baseline was excluded from this analysis.

### 2.4. Pregnancy-Unique Quantification of Emesis and Nausea (PUQE Questionnaire)

The PUQE questionnaire with good reliability (0.85) and validity (0.95) was adopted to evaluate the severity of nausea and vomiting during pregnancy [20]. This questionnaire consists of three questions regarding vomiting frequency, nausea intensity, and retching intensity. The scores of these three questions were added up, with higher scores reflecting more severe symptoms. The maximum score is 15, and a total score of ≥13 can be considered as severe nausea and vomiting of pregnancy (NVP) [21].

### 2.5. Diagnostic Criteria for HG

The diagnostic criteria for HG in this study were based on established guidelines and previous studies [22,23,24], as follows:

1. Before 16 weeks of gestation, severe nausea and frequent vomiting impair normal eating and significantly limit daily activities [22].
2. Received medical attention and therapeutic measures for NVP [23].
3. A PUQE score of ≥13 points [23].
4. A weight loss of >5% of pre-pregnancy body weight due to nausea or vomiting [24].
5. Exclude other potential causes of vomiting, such as gastrointestinal or urinary tract infections, viral hepatitis, or pre-existing medical conditions [24].

### 2.6. Assessment of Other Variables

Demographic data, including age, educational attainment, employment status, annual household income, smoking, and alcohol consumption, as well as details of pregnancy-related information like parity, gestational week and nutritional supplement usage were gathered through questionnaires and electronic health records. Height and weight were measured by trained investigators using a JUMPER weight and height meter (Smart Obstetrics Version 2.0) during the first obstetric examination. Body mass index (BMI) was calculated as weight (kg) divided by squared height (m^2^). The International Physical Activity Questionnaire Short Form (IPAQ) [25], which consists of seven questions inquiring about the frequency and duration of vigorous and moderate physical activities, walking and sedentary behavior over the preceding week, and enables the calculation of average daily metabolic equivalent (MET-min/week).

### 2.7. Statistical Analysis

Normally distributed variables were reported as mean ± standard deviation, while non-normally distributed variables were presented as median and interquartile range M (P75-P25) and Categorical variables were expressed as *n* (%). The analysis was conducted on the entire study population (*N* = 2418), including both HG cases (*n* = 212) and non-HG cases (*n* = 2206). The participants were stratified into quartiles (Q1–Q4) based on their energy-adjusted dietary intake of soy isoflavones. Descriptive statistics were employed to compare the general characteristics of the participants across these quartiles. Logistic regression analyses were used to explore the association between dietary soy isoflavones intake and the risk of HG, with the lowest intake group serving as the reference group. Odds ratio (OR) and 95% confidence interval (CI) were calculated for the other three groups. To account for multiple comparisons, the significance level was adjusted using the Bonferroni correction. The adjusted *p*-values are presented in the results section. Three regression models were fitted: Unadjusted Model was unadjusted; Partially Adjusted Model was adjusted for age, gestational week, parity, and total energy intake; and Fully Adjusted Model was further adjusted for physical activity, pre-pregnancy BMI, annual household income, educational level, employment status, smoking, alcohol consumption and the use of nutritional supplements [26]. Furthermore, the median values of each quartile were treated as continuous variables in logistic regression models to obtain *p*-values for trends in independent variables and outcome indicators. Restricted cubic spline models with 3 knots (at 25%, 50%, and 75% percentiles) were used to explore the dose-response association between dietary soy isoflavones intake and risk of HG.

Data collection was performed using the REDCap platform, with subsequent entry through the EpiData 3.0 software. Statistical analyses were conducted using R (version 4.4.0; R Foundation for Statistical Computing, Vienna, Austria). Data management and preprocessing were carried out using the *dplyr* package, while visualization was performed using *ggplot2*. Statistical significance was defined as a two-tailed *p* < 0.05.

## 3. Results

Among all 2418 pregnant women included in the study, 212 (8.8%) were diagnosed with HG. All statistical analyses, including logistic regression models assessing the association between dietary soy isoflavones intake and HG risk, were conducted based on the entire study population (*n* = 2418). The median intake of dietary soy isoflavones was 14.56 (interquartile range: 9.89–25.36) mg/day, with median intakes for the quartiles being 7.61, 11.93, 18.18 and 36.34 mg/day, respectively. Table 1 presents the general characteristics of the study population stratified by quartiles of dietary soy isoflavones intake. Supplementary Figure S1 illustrates the distribution of dietary soy isoflavones among all participants. Significant differences were observed across the various soy isoflavones intake groups in terms of age, gestational week, total energy intake, consumption of vegetables, fruits, meats, and seafood, as well as parity (Table 1); however, no significant differences were noted for pre-pregnancy BMI, physical activity, educational level, employment status, annual household income, use of nutritional supplements, smoking, or alcohol consumption. Supplementary Table S1 details the five major food groups contributing to soy isoflavones intake and their respective consumption levels. Legumes emerged as the predominant source of dietary isoflavones, contributing an average of 2.26 (interquartile range: 2.25–14.44) mg/d, which accounted for 37.54% of total dietary soy isoflavones intake, followed by tofu, bean sprouts, and soy milk. Non-legume foods, which contain minimal amounts of soy isoflavones, contributed only 3.52% of the total contribution.

Supplementary Table S2 compares the general characteristics between the HG (*n* = 212) and non-HG (*n* = 2206) groups. Notably, the HG group exhibited a higher gestational age and a lower intake of meat, fish and seafood in comparison to the non-HG group; however, the other variables showed no significant differences. Supplementary Table S3 details the intake of various food groups among all participants across quartiles of dietary soy isoflavones. The higher soy isoflavones intakes were significantly associated with higher intakes of legumes, tofu, bean sprouts, soymilk and non-legume foods containing soy isoflavones.

Table 2 summarizes the associations between covariates and the risk of HG. For each additional week of gestation, the risk of HG increased by 15%. Furthermore, when compared to pregnant women with the lowest meat intake (≤33.70 g/day), those with intakes of 33.70–77.30 g/day and ≥77.30 g/day exhibited a reduced risk of HG by 0.61-fold (95% CI: 0.44, 0.86) and 0.56-fold (95% CI: 0.39, 0.79), respectively. Additionally, pregnant women with the highest intake of fish and seafood demonstrated a 0.50-fold (95% CI: 0.35, 0.71) lower risk of HG compared to those with the lowest intake (≤20.10 g/day).

**Figure 1:**
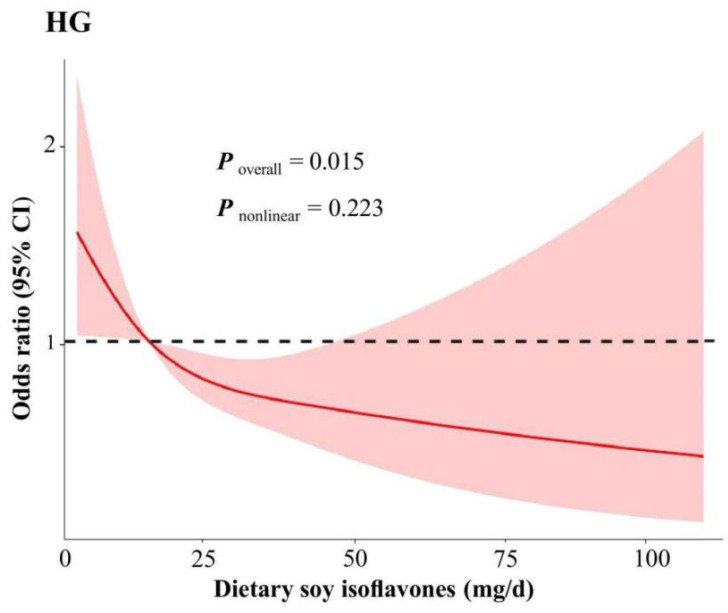
A multivariable-adjusted restricted cubic spline model was used to examine the association between dietary soy isoflavones intake and HG. Knots were positioned at the 25th, 50th, and 75th percentiles of the dietary soy isoflavones distribution. The solid red line represents the estimated odds ratio, while the red-shaded area indicates the 95% confidence interval. The binary logistic regression was adjusted for age, annual household income, educational level, occupation, physical activity, pre-pregnancy BMI, gestational week, parity, total energy intake, smoking, alcohol consumption, the use of nutritional supplements, intake of meats, fish and seafood, fruits and vegetables. BMI: body mass index. CI: confidence interval. HG: hyperemesis gravidarum.

Table 3 presents the associations between dietary soy isoflavones intake and HG. In the Unadjusted Model, higher intake of dietary soy isoflavones was associated with a significantly reduced risk of HG compared to lower intake (OR: 0.52, 95% CI: 0.34, 0.79; *P*_trend_ < 0.001). After adjusting for age, gestational week, parity, and total energy intake (Partially Adjusted Model), with higher intake of dietary soy isoflavones associated with a lower risk of HG (OR: 0.51, 95% CI: 0.33, 0.77; *P*_trend_ < 0.001). Although this association was attenuated in the Fully Adjusted Model, a significant association remained, indicating that higher dietary soy isoflavones intake was associated with a 44% reduction in risk of HG among pregnant women in quartile 4 compared to those in quartile 1 (OR: 0.56, 95% CI: 0.36, 0.88; *P*_trend_ = 0.012). The restricted cubic spline plot, adjusted for multiple variables (Figure 1), demonstrated a linear dose-response relationship, with HG risk decreasing as soy isoflavones intake increased (*P*_nonlinear_ = 0.223).

Supplementary Table S4 presents the associations between dietary soy isoflavones intake and HG risk after sequentially excluding specific food groups and components. Notably, when tofu (OR: 0.77, 95% CI: 0.62, 0.97; *P*_trend_ = 0.068), bean sprouts (OR: 0.79, 95% CI: 0.63, 1.00; *P*_trend_ = 0.282) and genistein (OR: 0.76, 95% CI: 0.61, 0.95; *P*_trend_ = 0.083) were excluded from the analysis, the association between dietary soy isoflavones and HG risk was no longer significant. In contrast, after controlling for other food groups and dietary components, the association between dietary soy isoflavones and HG risk remained statistically significant.

**Figure 2:**
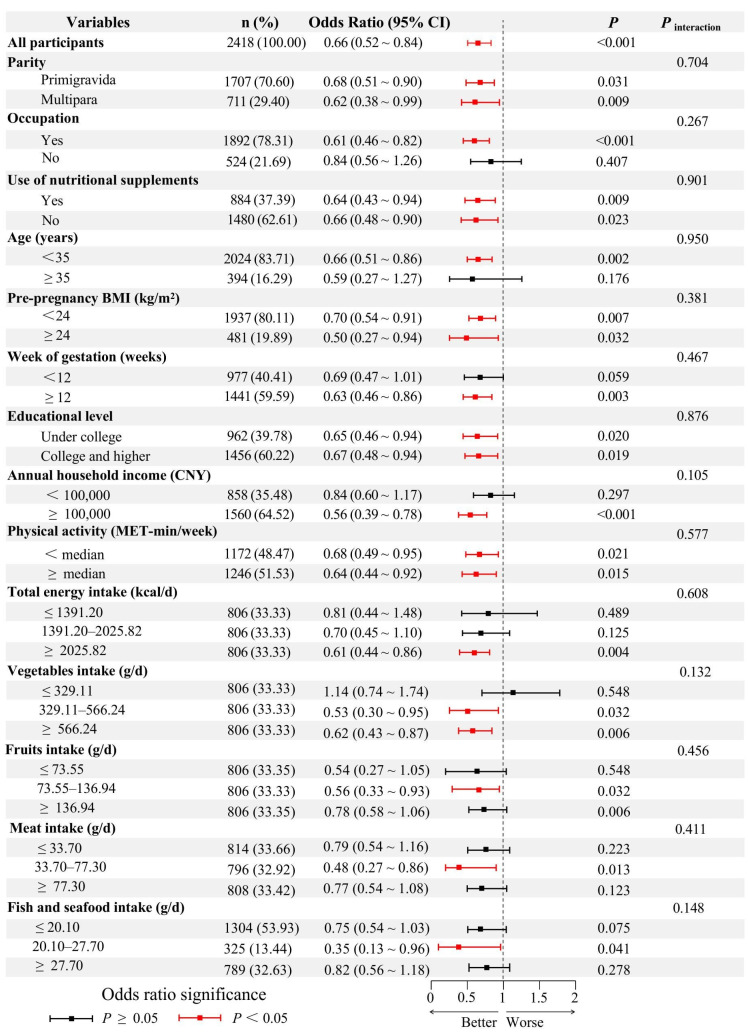
Subgroup analyses, adjusted for multiple variables, were conducted to explore the association between dietary soy isoflavones intake and HG. Given the linear nature of this association, dietary soy isoflavones were treated as a continuous variable (per standard deviation increase) in the subgroup analysis. The left section presents the variables, grouping categories, and sample sizes for each subgroup, along with the corresponding ORs and 95% CIs. A dashed vertical line represents an OR of 1, while squares denote the point estimates of the ORs, with horizontal lines illustrating the 95% CIs. Black indicates non-significant differences, whereas red highlights statistically significant differences. Due to the limited number of smokers and alcohol consumers in the sample, subgroup analysis for these variables were not performed. The binary logistic regression model was adjusted for age, gestational week, parity, total energy intake, physical activity, pre-pregnancy BMI, annual household income, educational level, occupation, smoking, alcohol consumption, the use of nutritional supplements, intake of meats, fish and seafood, fruits and vegetables. BMI: body mass index. OR: odds ratio. CI: confidence interval.

Figure 2 illustrates the subgroup analysis of the association between dietary soy isoflavones and the risk of HG. No significant differences were identified in the association between dietary isoflavones and HG risk across various subgroups, which included age, gestational week, parity, total energy intake, physical activity, pre-pregnancy BMI, annual household income, educational level, occupation, the use of nutritional supplements, intake of meats, fish and seafood, fruits and vegetables (*P*_intreaction_ > 0.05).

## 4. Discussion

To our knowledge, this is the first study have explored the association between dietary soy isoflavones intake and the risk of HG. In this cross-sectional study, we found that higher intake of dietary soy isoflavones was associated with a reduced risk of HG in the pregnant women after adjusting for all potential confounding factors. Notably, while our overall analysis identified this significant association, the subgroup analysis did not reveal significant interaction effects across various subgroups, such as age and gestational weeks. This indicates that the protective effect of soy isoflavones against HG is consistent across various subgroups, rather than being driven by specific population characteristics. These findings provide scientific evidence to the understanding of the association between dietary soy isoflavones intake and HG risk, offering theoretical support and new insights for prenatal nutritional guidance and early intervention strategies for HG.

The dietary intake of soy isoflavones in our study was 14.56 mg/d, which is moderate compared to other populations of Asian women. This figure comparable to those reported in two Korean studies (15.89 mg/day and 15.3 mg/day) [26,27], yet it is notably lower than the intake that reported in a Japanese study (37.4 mg/day) [28]. When contrasted with findings from studies involving Chinese women, our reported soy isoflavones intake was slightly higher than the levels documented by Zhang (8.07 mg/d) and Wei (9.4 mg/d) [29,30]. Thus, the dietary soy isoflavones intake observed in our study appears to be relatively representative. It is important to highlight that our study population predominantly consisted of individuals in early pregnancy, and the components included in the calculation of dietary soy isoflavones differ to some extent from those employed in the other studies, which may account for the observed discrepancies in the results.

We found significant differences in age, gestational weeks, total energy intake, and the consumption of vegetables, fruits, meat, and fish and seafood across the groups stratified by levels of soy isoflavones intake. In China, dietary habits during pregnancy are significantly shaped by traditional beliefs and cultural practices. For instance, many pregnant women tend to limit their consumption of cold or raw foods, including certain vegetables and seafood, guided by the traditional Chinese medicine (TCM) principles of “warming” and “cooling” foods. Additionally, soy-based products such as tofu and soy milk are commonly consumed due to their perceived benefits for maternal health. These cultural factors may partly explain the variations in dietary intake observed in our study. These variables may exert a partial influence on the consumption of soy isoflavones. A study conducted in Japan suggested that differences in age and gestational weeks might reflect dietary changes during pregnancy, which subsequently stimulate isoflavones consumption [31]. Additionally, variations in total energy intake and overall dietary composition—including vegetables, fruits, fish and seafood—may indicate differences in dietary patterns or health consciousness among individuals [32], which could indirectly influence soy isoflavones consumption. It is important to note that this association does not imply causation; rather, these factors may serve as potential confounders in the association between soy isoflavones and HG.

We found that gestational week, along with lower intake of meat and seafood, was positively correlated with HG. During early pregnancy, the gradual increase in estradiol and human chorionic gonadotropin (hCG) levels may significantly contribute to this association. Previous studies have shown that high levels of estradiol can reduce intestinal transit time and gastric emptying, which may lead to fluid accumulation in the body [33]. Additionally, hCG is known to stimulates gastrointestinal secretion, leading to increased fluid accumulation and subsequent intestinal distension. However, it must be noted that this cross-sectional study design precludes any definitive conclusions regarding. It is also possible that the nausea and vomiting experienced by pregnant women with HG may lead to a decrease in their consumption of foods such as meat and seafood. Despite the significant associations between gestational age, meat and seafood intake, and the risk of HG, these factors only partially explain the occurrence of HG. Moreover, we found that higher soy isoflavones intake was significantly associated with a lower risk of HG, and adjusting for potential variables did not subsequently diminish the protective effect of soy isoflavones intake.

Although one previous study examined the effect of soy-based supplementation (soy biscuits) on nausea and vomiting in HG patients [34], our study focuses on the habitual dietary intake of soy isoflavones in a general pregnant population. This population-based approach offers novel insights into the potential role of dietary soy isoflavones in HG prevention. We observed that higher soy isoflavones intake was associated with a lower HG risk. Our findings are consistent with those of a previous study, a national-level meta-analysis indicated that the incidence of NVP is related to high intakes of fats, carbohydrates, and meats, alongside a low intake of legumes [35]. Additionally, a prospective study conducted in the United Kingdom showed that the consumption of legumes among pregnant women experiencing NVP was lower compared to their non-NVP counterparts [36]. This potential association could involve intricate interactions between estrogen and its receptors, the anti-inflammatory and antioxidant properties of soy isoflavones, as well as the beneficial effects of high-quality proteins on physiological functions. However, the association between soy isoflavones and HG risk has not yet been investigated in the literature.

The mechanisms by which soy isoflavones counteract HG may include their estrogen-like effects, anti-inflammatory and antioxidant properties, and antibacterial activity. First, soy isoflavones exhibit structural similarities to estrogens [37]. Although their physiological effects in organism have not been fully elucidated, it is reported that they can bind to both α and β estrogen receptors, exhibiting dual characteristics of estrogenic and anti-estrogenic activity [38]. It means that soy isoflavones may function as estrogen agonists in individuals with low serum estrogen levels, while acting as estrogen antagonists in those with high estrogen levels [39]. HG usually manifests within weeks following the peak secretion of hormones from the placenta and corpus luteum, with the stimulation of certain endocrine hormones, including estrogen and progesterone, posted as a primary contributor to HG [40]. Therefore, the phytoestrogenic properties of soy isoflavones may reduce uterine exposure to circulating estrogens, potentially lowering the risk of HG.

Additionally, HG is considered as a process of oxidative stress. This is compounded by the increase production of reactive oxygen species (ROS) in the placenta due to heightened mitochondrial activity during early pregnancy [41]. The persistent vomiting and electrolyte disturbances caused by HG further aggravate the state of oxidative stress [42]. A case-control study involving 30 cases and 30 control indicated that pregnant women with HG exhibiting signs of oxidative imbalance [43]. An animal study demonstrated that SENCAR mice fed a diet containing 50 ppm or 250 ppm purified genistein for 30 days exhibited higher activities of catalase, glutathione reductase, and glutathione S-transferase in the small intestine compared to the control group [44]. Another study found that administration of a novel isoflavone supplement rich in daidzein significantly reduced the secretion of pro-inflammatory cytokines in Mono Mac 6 human macrophages and mesenteric lymph node cells derived from colitis-induced mice [45]. These notable anti-inflammatory and antioxidant properties of soy isoflavones may alleviate oxidative stress and intestinal inflammation induced by dietary or chemical factors, thereby exerting a protective effect on the gastrointestinal tract [46,47,48].

Furthermore, Studies have indicated that *Helicobacter pylori* (*Hp*) is a risk factor for NVP [49], and the severity of nausea and vomiting in HG pregnant women exhibits a dose-response relationship with *Hp* infection [50]. A meta-analysis also supports that *Hp* infection can increase the risk of HG. However, some studies have shown that soy isoflavones can inhibit the growth of *Hp* by interfering with the activity of topoisomerase II [51,52]. This antimicrobial property of soy isoflavones suggests that they may, to some extent, reduce the risk of HG.

Therefore, our findings suggest that higher soy isoflavones intake may be associated with a reduced risk of HG, potentially alleviating its clinical symptoms. However, the cross-sectional design prevents drawing definitive conclusions about causality. Sensitivity analysis revealed that after excluding tofu, bean sprouts, and genistein, the association between soy isoflavones intake and HG risk was no longer significant. These findings suggest that tofu, bean sprouts, and genistein may potentially contribute to the protective effects of soy isoflavones against HG. However, further studies are needed to confirm the individual effects of specific soy isoflavones components. Tofu is an excellent source of high-quality protein [53,54]. While the protein in tofu is relatively similar to that found in meat and dairy products, it does not contain cholesterol or saturated fatty acids [55]. Tofu may help reduce fat accumulation, enhance fat metabolism, and promote weight loss by modulating the expression of genes associated with appetite-suppressing factors [56]. A clinical study conducted in the United States found that a high-protein diet provided to pregnant women experiencing nausea and vomiting in early pregnancy could better alleviate these symptoms [57]. In addition, bean sprouts are rich in dietary fiber and various vitamins [58,59]. Studies have shown that elevated vitamin D levels are associated with a reduced risk of HG [60]. All these components may work synergistically with soy isoflavones to reduce the risk of HG. Furthermore, genistein, as a major soy isoflavones, may exert its effects through hormone regulation, antioxidant activity, and anti-inflammatory properties, which are likely key mechanisms in preventing HG [61,62]. These findings indicate that the protective effects of soy isoflavones on HG risk are not uniformly distributed across all sources or components but may depend on specific foods or individual soy isoflavones components. Further research is needed to explore the role of specific soy isoflavones components and their synergistic interactions with other dietary factors to better understand their contribution to HG prevention.

This study has several strengths, notably being the first to investigate the association between dietary soy isoflavones intake and the risk of HG. Furthermore, our research was conducted in China and included a large sample of pregnant women, which better reflects the dietary habits of pregnant women in this region. Additionally, in terms of the calculation of dietary soy isoflavones, we selected three substances-daidzein, genistein, and glycitein-to estimate soy isoflavones intake, which can accurately represent the dietary intake of soy isoflavones among pregnant women [63]. However, this study also has certain limitations. First, legumes and their products are diverse and serve as a primary source of high-quality protein for Chinese residents. However, we did not account for cooking methods, it was impossible to obtain an accurate intake of soy isoflavones. Therefore, we used the average value for calculation. Second, the dietary soy isoflavones intake was evaluated using the FFQ, which may result in dietary intake misclassification. Third, although we have used a multivariate model to adjust for possible confounding factors, there may still be some potential confounding factors that have not been included. Fourth, due to the inherent limitations of a cross-sectional design, we cannot make definitive causal inferences. Therefore, future prospective studies are necessary to confirm our findings. In addition to epidemiological validation, future research should also explore the clinical implications of soy isoflavones for HG management. Interventional studies could evaluate whether dietary supplementation with specific soy isoflavones components (e.g., genistein or daidzein) can alleviate HG symptoms or modulate hormone levels associated with nausea and vomiting. Moreover, examining the interaction between soy isoflavones intake and gut microbiota composition may provide new insights into the gastrointestinal mechanisms underlying HG. These clinical approaches could help translate our findings into practical dietary recommendations for pregnant women at risk of HG. Finally, since the study participants were primarily pregnant women from the northwest region of China, the generalizability of the results may be limited.

## 5. Conclusions

In this cross-sectional study, we observed an inverse association between dietary soy isoflavones intake and the risk of HG. We advocate for increased consumption of legumes and legume-derived products containing soy isoflavones among pregnant women, as these compounds may serve as protective factors against the development of HG. These findings may provide epidemiological evidence for the prevention of HG and the formulation of related policies in the future. Nevertheless, prospective studies are still needed to validate our findings.

## Figures and Tables

**Table 1:** General characteristics of the study population stratified by the quartiles of dietary soy isoflavones intake.

Characteristic	Quartiles of Dietary Soy Isoflavones Intake				*p*
	Q1 (Low)	Q2	Q3	Q4 (High)	
No. of participants (*n*)	605	604	604	605	
Age (years), mean ± SD	30.7 ± 3.4	31.4 ± 3.7	31.4 ± 3.4	31.2 ± 3.3	0.001
Pre-pregnancy BMI (kg/m^2^), mean ± SD	21.8 ± 3.3	21.8 ± 3.2	21.9 ± 3.4	21.9 ± 3.9	0.932
Week of gestation (weeks), median (IQR)	12.0 (8.3, 12.5)	12.2 (10.2, 12.7)	12.2 (10.2, 12.7)	12.0 (9.3, 12.7)	<0.001
Physical activity (MET-min/week), median (IQR)	17.26 (11.31, 19.64)	17.27 (11.10, 19.33)	17.27 (11.43, 19.60)	17.27 (11.31, 19.72)	0.879
Total energy intake (kcal/d), median (IQR)	1863.13 (1428.04, 2313.11)	1318.75 (1015.86, 1871.18)	1560.38 (1234.65, 2044.45)	1915.35 (1501.38, 2557.71)	<0.001
Vegetables intake (g/day), median (IQR)	325.85 (222.92, 478.39)	342.32 (225.98, 510.91)	470.08 (329.48, 652.87)	629.29 (456.79, 914.70)	<0.001
Fruits intake (g/day), median (IQR)	80.68 (48.43, 131.72)	80.89 (49.66, 132.75)	106.97 (70.08, 171.11)	142.45 (92.63, 220.95)	<0.001
Meat intake (g/day), median (IQR)	54.70 (28.50, 90.90)	43.70 (26.10, 77.30)	59.70 (31.10, 95.90)	77.30 (38.70, 130.50)	<0.001
Fish and seafood intake (g/day), median (IQR)	20.10 (15.10, 30.30)	20.10 (15.10, 27.70)	20.10 (15.10, 41.00)	27.70 (20.10, 42.90)	<0.001
Occupation (yes), *n* (%)	460 (76.16)	476 (78.81)	467 (77.45)	489 (80.83)	0.235
Use of nutritional supplements (yes), *n* (%)	210 (35.41)	221 (37.39)	215 (36.82)	238 (39.93)	0.437
Smoking (yes), *n* (%)	25 (4.13)	20 (3.31)	17 (2.81)	16 (2.64)	0.460
Alcohol consumption (yes), *n* (%)	29 (4.79)	22 (3.64)	16 (2.65)	14 (2.31)	0.073
Educational level, *n* (%)					0.164
Under college	258 (42.64)	227 (37.58)	227 (37.58)	250 (41.32)	
College and higher	347 (57.36)	377 (62.42)	377 (62.42)	355 (58.68)	
Annual household income, CNY, *n* (%)					0.959
<100,000	217 (35.87)	218 (36.09)	213 (35.26)	210 (34.71)	
≥100,000	388 (64.13)	386 (63.91)	391 (64.74)	395 (65.29)	
Parity, *n* (%)					0.045
Primigravida	449 (74.21)	405 (67.05)	420 (69.54)	433 (71.57)	
Multipara	156 (25.79)	199 (32.95)	184 (30.46)	172 (28.43)	

Note. *p*-values were derived from a one-way ANOVA test or Kruskal-Wallis test (continuous variables) or Chi-squared test (categorical variables). BMI: body mass index. MET: metabolic equivalent; SD: standard deviation; IQR: interquartile range.

**Table 2:** Association between covariates in the model with hyperemesis gravidarum.

Variables	Participants (%)	OR (95% CI)	*p*
Age (continuous)	2418 (100.00)	0.98 (0.93, 1.02)	0.297
Week of gestation (continuous)	2418 (100.00)	1.15 (1.07, 1.24)	<0.001
Physical activity (continuous)	2418 (100.00)	1.01 (0.98, 1.03)	0.449
Pre-pregnancy BMI ^a^ (<24 kg/m^2^/≥24 kg/m^2^)	1937/481 (80.11/19.89)	0.99 (0.70, 1.42)	0.975
Educational level ^b^ (Under college/College and higher)	962/1456 (39.78/60.22)	0.85 (0.64, 1.13)	0.261
Annual household income ^c^ (<100,000 CNY/≥100,000 CNY)	858/1560 (35.48/64.52)	0.84 (0.63, 1.12)	0.243
Parity ^d^ (Primigravida/Multipara)	1707/711 (70.60/29.40)	0.97 (0.71, 1.32)	0.833
Use of nutritional supplements e (no/yes)	884/1480 (37.39/62.61)	1.11 (0.83, 1.50)	0.475
Smoking ^e^ (no/yes)	2340/78 (94.32/5.68)	1.20 (0.57, 2.52)	0.637
Alcohol consumption e (no/yes)	2337/81 (96.65/3.35)	0.53 (0.19, 1.47)	0.223
Total energy intake			
≤1391.20 kcal/day	806 (33.33)	1.00 (Ref)	
1391.20–2025.82 kcal/day	806 (33.33)	1.09 (0.76, 1.55)	0.648
≥2025.82 kcal/day	806 (33.33)	1.20 (0.85, 1.69)	0.296
Vegetables intake			
≤329.11 g/day	806 (33.33)	1.00 (Ref)	
329.11–566.24 g/day	806 (33.33)	0.81 (0.55, 1.09)	0.140
≥566.24 g/day	806 (33.33)	0.81 (0.58, 1.14)	0.228
Fruits intake			
≤73.55 g/day	806 (33.33)	1.00 (Ref)	
73.55–136.94 g/day	806 (33.33)	0.79 (0.56, 1.12)	0.189
≥136.94 g/day	806 (33.33)	0.90 (0.64, 1.26)	0.547
Meat intake			
≤33.70 g/day	814 (33.66)	1.00 (Ref)	
33.70–77.30 g/day	796 (32.92)	0.61 (0.44, 0.86)	0.004
≥77.30 g/day	808 (33.42)	0.56 (0.39, 0.79)	0.001
Fish and seafood intake			
≤20.10 g/day	1304 (53.93)	1.00 (Ref)	
20.10–27.70 g/day	728 (13.44)	0.71 (0.46, 1.11)	0.140
≥27.70 g/day	189 (32.63)	0.50, (0.35, 0.71)	<0.001

Note. ^a^ Reference group: <24 kg/m^2^. ^b^ Reference group: Under college. ^c^ Reference group: <100,000 CNY. ^d^ Reference group: Primigravida. ^e^ Reference group: no. OR: odds ratios; CI: confidence interval; BMI: body mass index.

**Table 3:** Association between dietary soy isoflavones intake and hyperemesis gravidarum risk.

	Quartiles of Dietary Soy Isoflavones Intake (OR, 95% CI)				*P*_trend_ ^a^
	Q1 (Low)	Q2	Q3	Q4 (High)	
Median (IQR), mg/d	7.60 (5.89, 8.77)	11.93 (10.87, 12.99)	18.18 (16.19, 21.65)	36.34 (30.15, 46.69)	
Case/total	69/605	53/604	52/604	38/605	
Unadjusted Model	1.00 (Ref)	0.74 (0.51, 1.09)	0.73 (0.50, 1.07)	0.52 (0.34, 0.79)	<0.001
Partially Adjusted Model	1.00 (Ref)	0.68 (0.46, 1.01)	0.69 (0.47, 1.01)	0.51 (0.33, 0.77)	<0.001
Fully Adjusted Model	1.00 (Ref)	0.76 (0.47, 1.15)	0.77 (0.52, 1.16)	0.56 (0.36, 0.88)	0.012

Note. ^a^ *P*_trend_ was evaluated by assigning the median values of each quartile of dietary intake to a continuous variable. Q1 and Q4 represent the lowest and highest quartile groups of dietary soy isoflavones intake. Unadjusted Model: no adjustments were made for confounding variables. Partially Adjusted Model: adjusted for age, gestational week, parity, and energy intake. Fully Adjusted Model: further adjusted for physical activity, pre-pregnancy BMI, annual household income, educational level, occupation, smoking, alcohol consumption, the use of nutritional supplements, intake of meats, fish and seafood, fruits, and vegetables.

## Data Availability

Due to data protection restrictions, the data are only available on request from the corresponding authors of the data presented in this study.

## Supplementary Materials

The following supporting information can be downloaded at: https://www.mdpi.com/article/10.3390/nu17071282/s1, Figure S1: Distribution of the dietary soy isoflavones intake among the study participants (*n* = 2418); Table S1: Contribution of different food groups to dietary soy isoflavones among participants; Table S2: General characteristics of hyperemesis gravidarum and non-hyperemesis gravidarum groups; Table S3: Different food groups intake of study participants across quartiles of dietary soy isoflavones; Table S4: Adjusted associations between dietary soy isoflavones (excluding each food groups and components individually) and the risk of hyperemesis.
