# Supplementary material for: Association Between Dietary Soy Isoflavones Intake and the Risk of Hyperemesis Gravidarum: A Cross-Sectional Study in Chinese Pregnant Women
Source: Nutrients. 2025 Apr 7;17(7):1282. doi: 10.3390/nu17071282 (PMC11990840; doi:10.3390/nu17071282)
Supplement: Supplementary file 1 [file nutrients-17-01282-s001.zip › nutrients-3552914-supplementary.pdf]

nutrients

MDPI

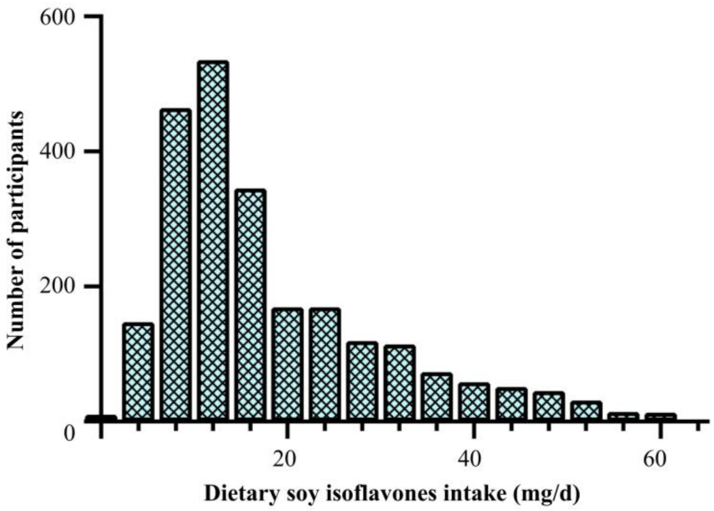

Figure S1. Distribution of the dietary soy isoflavones intake among the study participants (n = 2418).

Table S1. Contribution of different food groups to dietary soy isoflavones among participants.

Food Groups	Rank	Contribution (%)	Intake (mg/day)
Dietary soy isoflavones		100	14.56 (9.89, 25.36)
Legumes	1	37.54	2.26 (2.25, 14.44)
Tofu	2	22.05	2.33 (1.48, 5.51)
Bean sprouts	3	21.99	1.74 (1.74, 3.71)
Soy milk	4	14.90	1.35 (0.63, 4.06)
Non-legume foods	5	3.52	0.54 (0.36, 0.80)

Note. Data are median and interquartile range [M (P75-P25)].

Table S2. General characteristics of hyperemesis gravidarum and non-hyperemesis gravidarum groups.

Characteristic	All	Non-HG	HG	*P*
No of participants, n (%)	2418 (100.00)	2206 (91.23)	212 (8.77)	
Age (years), mean± SD	31.2± 3.4	31.2± 3.4	30.9± 3.5	0.315
Pre-pregnancy BMI, (kg/m2), mean± SD	21.84± 3.46	21.85± 3.49	21.64± 2.78	0.888
Week of gestation (weeks), median (IQR)	12.00 (9.50, 12.67)	12.00 (9.33, 12.67)	12.33 (11.50, 12.67)	＜0.001
Physical activity (MET-min/week), median (IQR)	17.27, (11.31, 19.46)	17.27 (11.31, 19.64)	17.27, (11.31, 19.76)	0.518
Total energy intake (kcal/d), median (IQR)	1693.80, (1250.36, 2223.27)	1262.22 (940.37, 1664.45)	1314.00 (926.10, 1699.12)	0.554
Vegetables intake (g/day), median (IQR)	433.41, (284.27, 651.05)	436.92 (286.63, 653.30)	405.08 (268.27, 631.59)	0.126
Fruits intake (g/day), median (IQR)	101.08, (60.51, 167.53)	100.88 (60.78, 16.29)	101.49 (54.42, 175.01)	0.351
Meat intake (g/day), median (IQR)	59.70, (31.10, 100.90)	59.70 (31.10, 100.90)	38.70 (23.50, 79.90)	＜0.001
Fish and seafood intake (g/day), median (IQR)	20.10, (15.10,35.30)	20.10 (15.10, 42.90)	20.10 (15.10, 27.70)	＜0.001
Occupation (yes), n (%)	1892 (78.31)	1731 (78.54)	161 (75.94)	0.383
Use of nutritional supplements (yes), n (%)	884 (37.39)	803 (37.18)	81 (39.71)	0.496
Smoking (yes), n (%)	78 (3.23)	70 (3.17)	8 (3.77)	0.547
Drinking (yes), n (%)	81 (3.35)	77 (3.49)	4 (1.89)	0.314
Educational level, n (%)				0.271
Under college	962 (39.78)	870 (39.44)	92 (43.40)	
College and higher	1456 (60.22)	1336 (60.56)	120 (56.60)	
Annual household income (CNY), n (%)				0.260
＜100,000	858 (35.48)	775 (35.13)	83 (39.15)	
≥100,000	1560 (64.52)	1431 (64.87)	129 (60.85)	
Parity, n (%)				0.875
Primigravida	1707 (70.60)	1556 (70.53)	151 (71.23)	
Multipara	711 (29.40)	650 (29.47)	61 (28.77)	

Note. *P*-values were derived from an independent-sample t-test or Wilcoxon's rank sum test (continuous variables) or Chi-squared test (categorical variables). SD: standard deviation. IQR: interquartile range. BMI: body mass index. HG: hyperemesis gravidarum.

Table S3. Different food groups intake of study participants across quartiles of dietary soy isoflavones.

Food Groups	Quartiles of Energy-Adjusted Dietary Soy Isoflavones Intake				P
	Q1 (Low)	Q2	Q3	Q4 (High)	
Legumes (g/d), median (IQR)	6.70 (1.70, 6.70)	6.70 (4.20, 10.50)	12.40 (6.70, 22.30)	42.65 (24.80, 50.85)	<0.001
Tofu (g/d), median (IQR)	13.40 (8.40, 21.00)	16.00 (13.40, 28.60)	28.60 (21.00, 57.20)	57.20 (28.60, 85.80)	<0.001
Bean sprouts (g/d), median (IQR)	6.70 (1.70, 6.70)	6.70 (6.70, 14.30)	14.30 (6.70, 14.30)	14.30 (6.70, 42.90)	<0.001
Soymilk (g/d), median (IQR)	16.75 (16.75, 35.75)	35.75 (16.75, 35.75)	107.25 (35.75, 107.25)	107.25 (107.25, 196.50)	<0.001
Non-legume foods (g/d), median (IQR)	416.45 (301.16, 617.00)	435.99 (297.70, 648.65)	600.42 (436.78, 1124.74)	803.64 (582.32, 1133.74)	<0.001

Note. IQR: interquartile range. P-values were derived from Kruskal-Wallis test.

Table S4. Adjusted associations between dietary soy isoflavones (excluding each food groups and components individually) and the risk of hyperemesis gravidarum.

	Quartiles of Dietary Soy Isoflavones (OR, 95% CI)				P_{trend}	Per SD Increase
	Q1 (Low)	Q2	Q3	Q4 (High)		
Dietary soy isoflavones	1.00 (Ref)	0.76 (0.47, 1.15)	0.77 (0.52, 1.16)	0.56 (0.36, 0.88)	0.012	0.66 (0.52, 0.84)
Except legumes	1.00 (Ref)	0.68 (0.44, 1.05)	0.88 (0.58, 1.32)	0.35 (0.55, 0.88)	0.028	0.68 (0.51, 0.90)
Except tofu	1.00 (Ref)	0.65 (0.43, 0.99)	0.63 (0.42, 0.96)	0.61 (0.40, 0.94)	0.068	0.77 (0.62, 0.97)
Except bean sprouts	1.00 (Ref)	0.57 (0.37, 0.89)	0.77 (0.51, 1.16)	0.69 (0.45, 1.05)	0.282	0.79 (0.63, 1.00)
Except soymilk	1.00 (Ref)	0.73 (0.48, 1.12)	0.79 (0.52, 1.18)	0.55 (0.35, 0.87)	0.020	0.72 (0.55, 0.94)
Except non-legume foods	1.00 (Ref)	0.62 (0.41, 0.95)	0.77 (0.51, 1.14)	0.56 (0.36, 0.87)	0.034	0.72 (0.56, 0.93)
Except daidzein	1.00 (Ref)	0.56 (0.36, 0.88)	0.69 (0.45, 1.05)	0.56 (0.36, 0.87)	0.036	0.74 (0.89, 0.94)
Except genistein	1.00 (Ref)	0.57 (0.36, 0.89)	0.68 (0.44, 1.06)	0.61 (0.39, 0.95)	0.083	0.76 (0.61, 0.95)
Except glycitein	1.00 (Ref)	0.63 (0.42, 0.96)	0.78 (0.53, 1.12)	0.54 (0.35, 0.85)	0.024	0.72 (0.56, 0.93)

Note. Q1 and Q4 represent the lowest and highest quartile groups of dietary soy isoflavones. Model was adjusted for age, gestational week, parity, total energy intake, physical activity, pre-pregnancy body mass index, annual household income, educational level, occupation, smoking, alcohol consumption, the use of nutritional supplements, intake of meats, fish and seafood, fruits and vegetables. OR: odds ratio. SD: standard deviation.
